# Merging art with science: the reflex hammer meets the algorithm

**DOI:** 10.1017/S1092852926101278

**Published:** 2026-10-10

**Authors:** Jagannadha Avasarala

**Affiliations:** Department of Neurology, University of Kentucky Medical Center, USA

## Abstract

The neurological examination is a carefully choreographed sequence of observations and elicited responses that transforms clinical signs into meaningful data, providing the trainee with a framework for formulating a possible diagnosis. This art form of examination still lives on among a few masters of the craft, but the precision of digitized data points is gaining ground, as it should.

## The past, and the future

The neurological examination is a carefully choreographed sequence of observations and elicited responses that transforms clinical signs into meaningful data, providing the trainee with a framework for formulating a possible diagnosis. This art form of examination still lives on among a few masters of the craft, but the precision of digitized data points is gaining ground, as it should.

Neurology is uniquely positioned to lead the technological revolution in medicine because we can integrate our findings with technology that has precision. By integrating automated pupillometry, optical coherence tomography, digital force sensors, and unsupervised deep learning frameworks—for example—the field is ideally placed to usher in data-driven phenotypic signatures. However, quantitative tools are not without their downsides, such as calibration requirements for devices, device variability, algorithmic bias, cost, accessibility, and the need for validation across diverse clinical populations. It is important to recognize that reproducibility alone does not guarantee diagnostic validity or clinical utility. A digital device may produce a highly reproducible number, but that number must be interpreted in the clinical context and supported by normative data, test–retest reliability, sensitivity/specificity, meaningful thresholds, and evidence that it improves diagnosis, prognosis, or treatment decisions.

## The relative afferent pupillary defect

Pupillary light reactivity testing using automatic pupillometry is more precise and reproducible.^1^ A cornerstone of the neuro-ophthalmologic exam, the presence of a relative afferent pupillary defect (RAPD) is the *raison d’être* for further testing that can distinguish anterior visual pathway lesions from migraine or functional visual loss. The “swinging flashlight test (SFT)” as currently deployed to elicit RAPD is highly subjective. It depends on multiple factors—the light source intensity, the ambient room lighting, the speed of the “swing,” and the ability of the human eye to perceive subtle escape in a pupillary cycle—all of which are unstandardized. Since clinical evaluation is poor at detecting subtle RAPDs, a missed diagnosis of a lesion in the anterior visual pathway may be the result. Conversely, over-interpreting pupillary hippus as a defect can trigger neuroimaging and invasive workups. Automated pupillometry has demonstrated superior sensitivity to the traditional SFT for detecting RAPD. In a clinical study of patients with acute vision loss, 50% of RAPDs detected by automated pupillometry were missed by the SFT, and when results were discordant, physicians were significantly more likely to pursue workup of optic nerve disease based on pupillometry findings (61% versus 18%, *P* = 0.008).^2^ A validation study of a portable computerized pupillometer demonstrated strong correlation with expert clinical grading of RAPDs (*r* = 0.81, *P* < 0.0001) and distinguished clinically significant RAPDs (≥0.5 LU) with an Area Under the Curve (AUC) of 0.98 (95% CI 0.95–1.00), supporting its role as an objective screening tool in optic neuropathies.^3^ Ultrasound pupillometry (UP) has also shown the ability to detect subclinical optic nerve damage in patients with multiple sclerosis (MS). As well, UP sharply discriminated between acute optic neuritis (ON) eyes (*n* = 9) and healthy control (HC) eyes (*n* = 100, Area Under the Curve or AUC = 1, 95% CI: 1; 1). UP detected an RAPD in 5/31 (16%) patients with MS without prior ON who had not exhibited an RAPD during the SFT.^4^ Additionally, automated pupillometry outperforms manual penlight examination by providing standardized, quantitative, and highly reproducible measurements of pupillary reactivity that minimize the inter-observer variability inherent to subjective assessment.^1^

## Deep tendon reflexes: a study in subjectivity

Deep tendon reflexes (DTRs) are presented as binary inputs into the diagnostic algorithm: brisk, normal, or diminished. Yet they are affected by hammer type, striking angle, wrist biomechanics, patient relaxation and position (recumbent versus sitting up), or the examiner’s expectations. The DTRs exhibit inter- and intra-observer differences.^5^

The patellar reflex tested by a neuromuscular specialist may identify subtle asymmetries or reflex extinction that others might miss. Even the grading scale—ostensibly a five-point system—has no universally agreed-upon anchors—what differentiates 3+ from 4+, or when exactly does clonus begin? We describe reflexes numerically, but the numbers are subjective judgments.

## Sensory testing: precision without precision tools

Light touch is commonly tested with a wisp of cotton, but “cotton” is an undefined reagent. Is it the end of a cotton-tipped applicator, a piece of gauze, a feather, or a finger lightly brushing the skin? Each method produces a different afferent stimulus, applied with varying force and velocity. Asking the patient’s response is shaped by the examiner’s phrasing. “Does this feel different?” is not the same as “Tell me when you feel this,” as patients may amplify or downplay differences based on attention or fatigue. Examining for pinprick adds the variability of broken safety pins and vague instructions on “sharp” versus “dull.” Temperature testing is rarely performed with standardized material—clinicians often use a tuning fork or their hand—none of which delivers a reproducible thermal gradient. In clinical trials, sensory endpoints are notoriously noisy, requiring large sample sizes to overcome the lack of standardized instrumentation.

## Motor strength: a semi-quantitative art

Manual muscle testing is perhaps the most consequential and yet the least objective component of the examination. The widely used Medical Research Council (MRC) scale appears robust but possesses significant subjectivity.^6^ Strength grading relies on the examiner’s force, leverage, hand size, fatigue, and ability to stabilize joints. A “5/5” from one examiner may be a “4+” for another. Ratings vary over time depending on expectations or the patient’s performance during earlier parts of the exam.

In neuromuscular clinics, subtle asymmetries or fatigability patterns are identified because examiners are trained to look for them. In other settings, these findings may be missed entirely. The same patient, on the same day, can be labeled as having “normal strength” or “mild weakness” at different time points.

## Clinical consequences: variability masquerading as biology

The consequences of a subjective examination are wide-ranging and economically significant. Inconsistent findings in reflexes or muscle strength can lead to overdiagnosis of peripheral neuropathy, underdiagnosis of myelopathy, or misattribution of symptoms to functional disorders. For instance, documentation of “normal strength” may delay appropriate neurological referral when the patient has weakness in a muscle or muscle groups. Conversely, a specialist who perceives subtle weakness may trigger an extensive, high-cost workup. The patient’s trajectory depends as much on the examiner as on the disease. Clinical trials of neuropathy, myasthenia gravis, and MS are particularly affected when subjective elements are used as inclusion criteria or outcome measures.

## The way forward: quantifiable neurology

A modernized neurological examination must acknowledge its subjectivity and pursue quantification. Digital reflex hammers^7^ could measure peak acceleration and latency rather than relying on human interpretation. Force sensors embedded in gloves or handheld devices could quantify strength in Newtons rather than relying on MRC grades that conflate strength with examiner effort. Standardized sensory stimulators for quantitative sensory testing offer a gold standard for laboratory-based metrics.^8^

Video-based analysis and wearables capture gait, tone, tremor, and coordination more reproducibly than bedside evaluation^9^ and some metrics can be captured in real time.^10^ Digital biomarkers across non-communicable diseases remain in early validation stages. Critical barriers include limited validation in diverse populations, lack of interoperability, insufficient integration with electronic health records, and challenges in multimodal data synthesis. Machine-learning-supported pattern recognition could transform semiological patterns into quantifiable phenotypic signatures. Despite legitimate concerns about digitizing the neurological examination, the pursuit of quantitative metrics is inevitable; regardless of current reservations, the future of neurological assessment will increasingly be defined by such measures.
